# Bone marrow mesenchymal stem cells attenuate 2,5-hexanedione-induced neuronal apoptosis through a NGF/AKT-dependent pathway

**DOI:** 10.1038/srep34715

**Published:** 2016-10-05

**Authors:** Qingshan Wang, Guohua Sun, Chenxue Gao, Lina Feng, Yan Zhang, Jie Hao, Enjun Zuo, Cong Zhang, Shuangyue Li, Fengyuan Piao

**Affiliations:** 1 Department of Occupational and Environmental Health, Dalian Medical University, Dalian, Liaoning 116044, China; 2 Department of Clinical Laboratory, the First Affiliated Hospital of Dalian Medical University, Liaoning 116011, China; 3 College of Stomatology, Dalian Medical University, Dalian, Liaoning 116044, China; 4 Department of Nutrition and Food Safety, Dalian Medical University, Dalian, Liaoning 116044, China

## Abstract

Growing evidence suggests that the increased neuronal apoptosis is involved in *n*-hexane-induced neuropathy. We have recently reported that bone marrow-mesenchymal stem cells-derived conditioned medium (BMSC-CM) attenuated 2,5-hexanedione (HD, the active metabolite of *n*-hexane)-induced apoptosis in PC12 cells. Here, we explored the anti-apoptotic efficacy of BMSC *in vivo*. HD-treated rats received BMSC by tail vein injection 5 weeks after HD intoxication. We found that in grafted rats, BMSC significantly attenuated HD-induced neuronal apoptosis in the spinal cord, which was associated with elevation of nerve growth factor (NGF). Neutralization of NGF in BMSC-CM blocked the protection against HD-induced apoptosis in VSC4.1 cells, suggesting that NGF is essential for BMSC-afforded anti-apoptosis. Mechanistically, we found that the decreased activation of Akt induced by HD was significantly recovered in the spinal cord by BMSC and in VSC4.1 cells by BMSC-CM in a TrkA-dependent manner, leading to dissociation of Bad/Bcl-xL complex in mitochondria and release of anti-apoptotic Bcl-xL. The importance of Akt was further corroborated by showing the reduced anti-apoptotic potency of BMSC in HD-intoxicated VSC4.1 cells in the presence of Akt inhibitor, MK-2206. Thus, our findings show that BMSC attenuated HD-induced neuronal apoptosis *in vivo* through a NGF/Akt-dependent manner, providing a novel solution against *n*-hexane-induced neurotoxicity.

*N*-Hexane, an organic solvent, is widely used in various industrial processes, such as paints, varnishes, printing inks and shoe manufacturing. Chronic exposure of *n*-hexane in human and experimental animals induces central-peripheral neuropathy, which is mainly mediated by 2,5-hexanedione (HD), the toxic metabolite of *n*-hexane^1^. Clinic symptoms of *n*-hexane-induced neuropathy follow a stereotypic, temporal pattern that initially appears in the distal and gradually develops to the proximal end. Human exposure to *n*-hexane display numbness and tingling sensation in the toes and fingers, followed by progressive weakness particularly in distal legs^2^. HD intoxication in rats also showed sensor-motor deficits and abnormal electrophysiological changes, such as prolongation of distal latencies and slowing of nerve conduction velocities^3^. Morphological observation revealed that axon atrophy, segmental demyelination as well as distal axonal Wallerian degeneration are the main pathological damage^4^.

Although the mechanism is not fully elucidated, some evidence suggests that the increased apoptosis in neurons is involved in the pathophysiological changes induced by HD^5^,^6^,^7^,^8^. As early as 1996, Ogawa *et al.* detected apoptosis in cultured dorsal root ganglion neurons treated with a low concentration of HD^7^. We recently also observed increased number of TUNEL-positive cells, expressions of Bax and cytochrome C (Cyt C) as well as activity of caspase-3 in HD-intoxicated PC12 cells^9^. Furthermore, Wistar rats intoxicated with 200 or 400 mg/kg HD displayed disturbance of Bcl-2 and Bax expressions as well as caspase-3 activity in nerve tissues^6^, indicating the involvement of apoptosis *in vivo*. It is recognized that massive neuron apoptosis inevitably causes loss of nerve tissue, structure instability and compensatory fibrosis, all of which promote the development of HD-induced neuropathy^10^. We therefore hypothesized that suppressing neuronal apoptosis and promoting recovery of injured neurons should be an effective strategy to alleviate the neurotoxic effects associated with HD.

Bone marrow mesenchymal stem cells (BMSC) are adult stem cells with strong self-renewing and differentiation abilities^11^. Due to their anti-inflammatory and anti-apoptotic properties, BMSC gain much attention in recent years and have been exploited to treat neurodegenerative diseases. The improved outcome of BMSC transplantation was observed in multiple neurodegenerative disorders, including Alzheimer’s disease, Parkinson’s disease and Amyotrophic Lateral Sclerosis^12^. We have previously reported that conditioned medium prepared from BMSC (BMSC-CM) protects PC12 cells against HD-induced apoptosis^9^. However, the anti-apoptotic efficacy and underlying mechanisms of BMSC in HD-induced neuropathy remain unclear.

In this study, we extended our previous findings by showing that BMSC transplantation promoted neuronal survival and attenuated HD-induced apoptosis in the spinal cord in a rat model, even performed after onset of neuronal damage. Elevation of nerve growth factor (NGF) and subsequent activation of Akt signaling pathway were recognized to be responsible for BMSC-elicited protection. Our findings strongly suggest that BMSC attenuate HD-induced apoptosis through a NGF/Akt-dependent manner, which may provide a novel way for combating *n*-hexane-induced neuropathy.

## Results

### BMSC attenuates HD-induced neuronal apoptosis in the spinal cord of rats

To determine the anti-apoptotic efficacy of BMSC, an animal model of *n*-hexane-induced neuropathy was generated in SD rats by injecting HD (400 mg/kg, i.p) for 5 consecutive weeks (5 times per week). Consistent with previous reports^13^,^14^,^15^,^16^, exposure to HD produced progressive motor deficits as shown by gait abnormalities. On about week 2, the rats of treated groups showed slightly gait abnormality, i.e. tiptoe walking, hindlimb adduction (mean gait score = 1.71 ± 0.25). As intoxication went on, the symptoms aggregated progressively. On about week 5, almost all rats showed severely abnormal gait (foot splay, dragging hindlimbs and inability to rear; mean gait score = 3.78 ± 0.44 (data no shown). Quantitative analysis revealed 20.94% loss of neurons in the spinal cord at the same time point.

**Figure 1:**
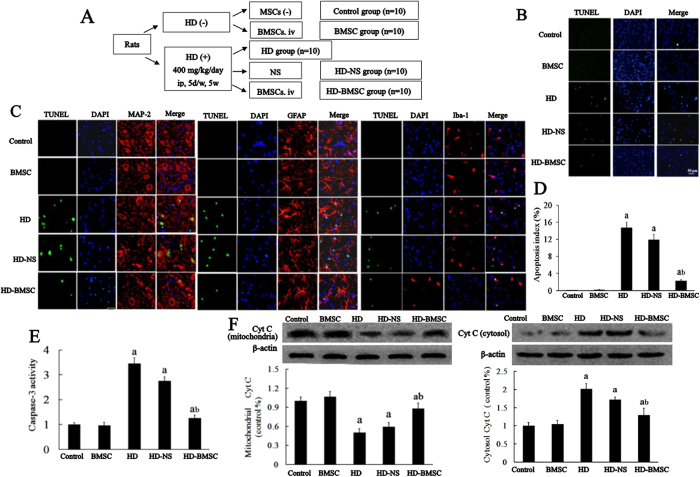
BMSC attenuates HD-induced neuronal apoptosis in the spinal cord of rats. (**A**) Schematic diagram of experimental design. SD rats were treated with HD (400 mg/kg, i.p) or saline for 5 consecutive weeks (5  times per week), and then were transplanted with 5 × 10^7^/kg BMSC by tail vein injection. Additional 5 weeks later, the spinal cords of rats were dissected. (**B**) TUNEL and DAPI staining were performed to detect apoptosis of cells in the spinal cord of rats. (**C**) MAP2, GFAP and Iba-1 were co-staining with Tunel, respectively, in the spinal cord of rats, and the representative images were shown. (**D**) TUNEL-positive cells were quantified. (**E**) The activity of caspase-3 in the spinal cord was detected using commercial caspase-3 activity detection kit. (**F**) The level of Cyt C was determined in both mitochondrial and cytosolic fractions of rats by Western blot and the density of blots was quantified (the full-length gels were shown in Supplementary Figure 2). Quantified data are shown as mean ± SEM. ^a^*p* < 0.05, compared with control group; ^b^*p* < 0.05, compared with HD group.

After 5 weeks of HD intoxication, rats received CFSE-labeled BMSC with 5 × 10^7^ cells/kg by tail vein injection and detected them in the spinal cord and sciatic nerve after 5 weeks of transplantation. Interestingly, we found that CFSE-labeled BMSC could be detected in the white matter and gray matter in the anterior horn of spinal cord by showing green fluorescent cells in these localizations (Supplementary Figure 1). After 5 weeks of BMSC transplantation, we investigated the effects of BMSC on HD-induced apoptosis in neurons in the spinal cord by TUNEL staining (Fig. 1A). As shown in Fig. 1B, almost no TUNEL-positive cells were observed in the spinal cord in both saline- and BMSC alone-treated rats. In contrast, HD-treated rats displayed an elevated number of TUNEL-positive cells in the spinal cord, indicating apoptosis occurs after HD intoxication. Further immunofluorescence staining against MAP2, GFAP and Iba-1 antibodies with TUNEL labeling revealed that HD-induced apoptosis occurred in neurons, astrocyte and microglia, respectively (Fig. 1C). Quantitative analysis revealed an about 15% increase of apoptotic cells in the spinal cord of HD-intoxicated rats compared with vehicle controls (Fig. 1D). Interestingly, BMSC transplantation markedly decreased HD-induced apoptosis by showing reduced number and percentage of TUNEL-positive cells in BMSC/HD-treated rats compared with HD alone group (Fig. 1D).

Mitochondrial signaling pathway is the major player in apoptosis, which is associated with release of Cyt C from mitochondria and subsequent activation of caspase-3^17^. Our results showed that compared with vehicle controls, the protein level of Cyt C in mitochondria was significantly decreased, while markedly increased in cytosolic fraction in the spinal cord of HD-intoxicated rats (Fig. 1E). HD intoxication also increased the activity of caspase-3 in the spinal cord (Fig. 1F). Consistent with reduction of TUNEL-positive cells, BMSC transplantation attenuated the release of Cyt C and activity of caspase-3 induced by HD, suggesting that BMSC blocked HD-induced mitochondria-dependent apoptosis (Fig. 1E,F).

### BMSC graft attenuates motor deficits and axon damage in HD-intoxicated rats

**Figure 2:**
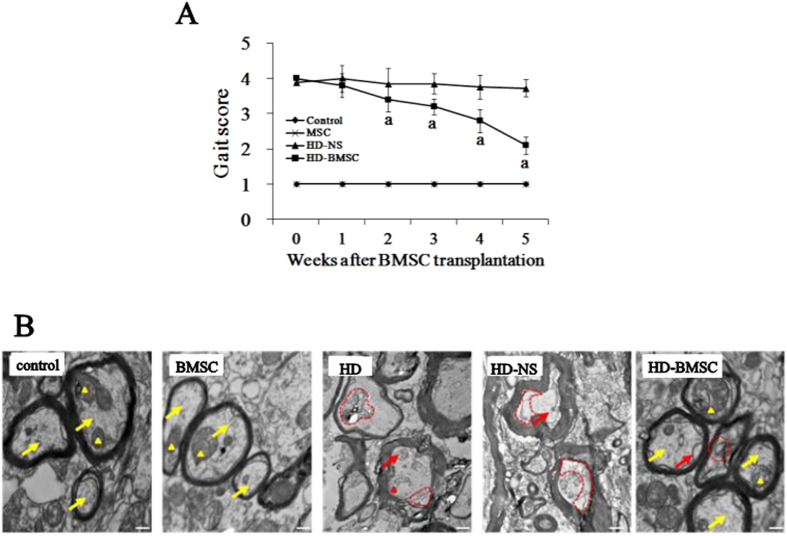
The changes of gait score and axon morphology of rats exposed to HD with or without BMSC. (**A)**, HD-intoxicated rats were transplanted with 5 × 10^7^/kg BMSC by tail vein injection. The gait score were recorded every week. (**B)** Electron microscope analysis was performed in the spinal cord of rats and the representative images were shown. ^a^*p* < 0.05, compared with HD-NS group.

To determine whether the anti-apoptotic effects displayed by BMSC were associated with functional recovery, we examined the gait behavior in HD-intoxicated rats with or without BMSC transplantation. We found that BMSC grafted rats displayed recovery of their motor function against HD-induced deficits as shown by a gradual decrease of gait score (Fig. 2A). Consistent with improvement of motor function, the axon damage induced by HD intoxication was also mitigated once HD-intoxicated rats were transplanted with BMSC (Fig. 2B).

### BMSC graft elevates NGF level in the spinal cord of HD-intoxicated rats

**Figure 3:**
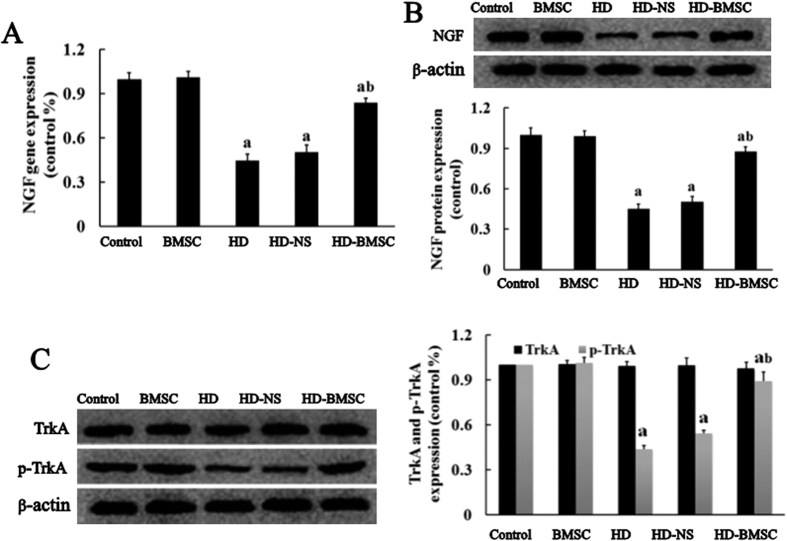
BMSC graft elevates the level of NGF in the spinal cord of HD-intoxicated rats. (**A**) The mRNA expression of NGF was assayed by real time RT-PCR. (**B**) Theprotein level of NGF was assayed by Western blot. (**C**) The levels of TrKA and p-TrKA were determined by Western blot and the density of blots was quantified (the full-length gels were shown in Supplementary Figure 3). ^a^*p* < 0.05, compared with control group; ^b^*p* < 0.05, compared with HD group.

Previous studies indicated that BMSC are capable of secreting neurotrophic factors, which play a key role in BMSC-elicited protection^18^,^19^. NGF is the best characterized member among the neurotrophic factors secreted from BMSC^20^ and promotes neuronal survival^20^,^21^. To determine whether the anti-apoptotic effect of BMSC was associated with release of NGF, we initially measured the mRNA and protein levels of NGF. Figure 3A showed that HD intoxication decreased the gene expression of NGF in the spinal cord of rats, which was significantly attenuated once HD-intoxicated rats were transplanted with BMSC. Consistent with increase of transcription level, the protein level of NGF was also recovered in BMSC-grafted rats (Fig. 3B). TrkA is well known to be the G-protein coupled receptor of NGF. Further study revealed that BMSC transplantation also reversed the reduced phosphorylation status of TrkA induced by HD in the spinal cord of rats (Fig. 3C), suggesting that elevated NGF stimulates the activation of its receptor.

### NGF is a key to mediate BMSC-afforded neuroprotection

**Figure 4:**
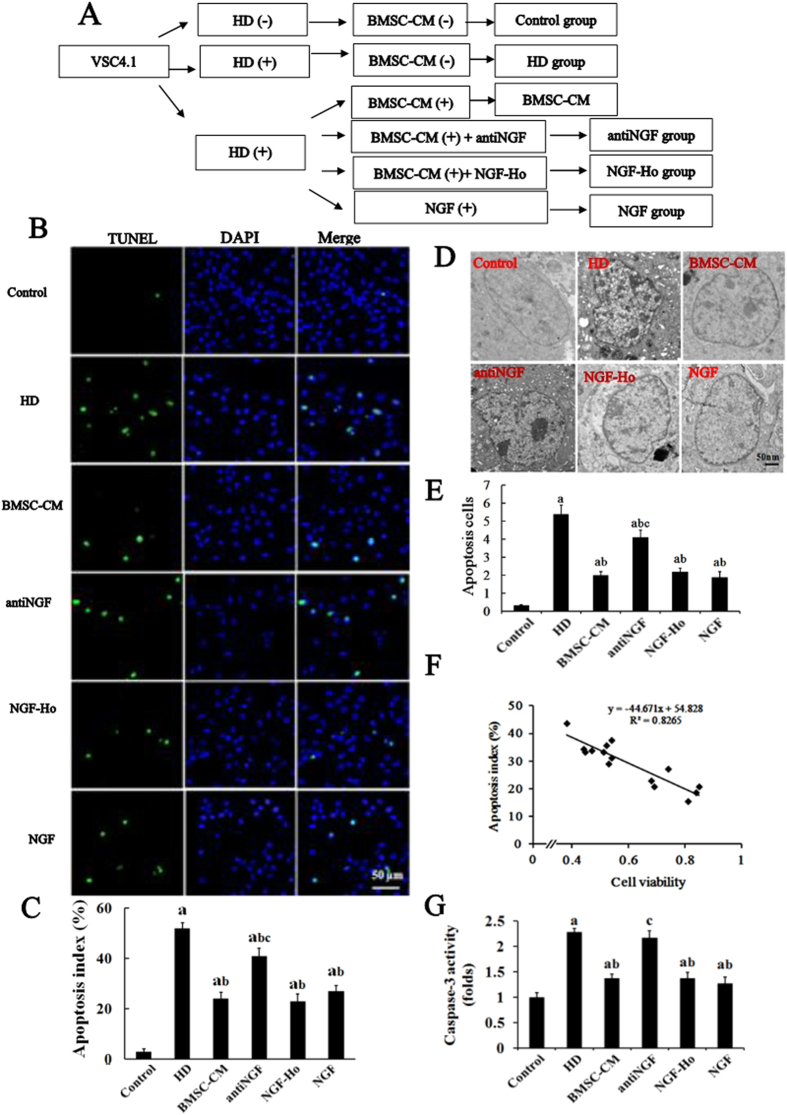
NGF is a key to mediate BMSC-afforded neuroprotection. (**A**) Schematic diagram of experimental design. VSC4.1 cells were treated with HD (25 mM) or saline for 24 h and then were treated with BMSC-CM (15%, v/v) or NGF (100 μM) in the presence or absence of anti-NGF antibody (10 μM) for additional 24 h. (**B**) TUNEL and DAPI staining were performed to detect apoptosis of cells in different groups. (**C**) The percentage of TUNEL-positive cells in different groups was quantified. (**D**) SEM was used to detect the morphological changes of cells in different groups. (**E**) Quantification of the morphological changed cells. (**F**) A linear regression analysis was performed between reduction of apoptosis and increase of neuronal survival. (**G**) The activity of caspase-3 in different groups was detected using commercial caspase-3 activity detection kit. Data represent mean ± SD for n = 3 sections per rat from n = 3 per group. ^a^*p* < 0.05, compared with control group; ^b^*p* < 0.05, compared with HD group; ^c^*p* < 0.05, compared with BMSC-CM group.

To further demonstrate the role of NGF in BMSC-afforded neuroprotection, we tested the impact of NGF neutralization on the anti-apoptotic effect of BMSC in an *in vitro* model of HD-induced apoptosis. We recently demonstrated that NGF released by BMSC accumulates in BMSC-CM and is capable of protecting PC12 cells against HD-induced apoptosis^9^. Consistent with previous report, the protective effect of BMSC-CM against HD-induced apoptosis was also observed in ventral spinal cord 4.1 (VSC4.1) cells, a cell line of dorsal motor neurons, by showing decreased number of TUNEL-positive cells and activity of caspase-3 compared with HD alone group (Fig. 4A–C,F). The anti-apoptotic efficacy of BMSC-CM was further confirmed by SEM analysis. As shown in Fig. 4D,E, the cells in HD alone-treated group displayed irregular shape, membrane curl and cytosolic vacuolation, all of which were significantly attenuated by BMSC and NGF treatment.

To determine the relationship between the reduction in TUNEL-positivity and increase in survival of neurons, a linear regression analysis between reduction of apoptosis by BMSC-CM and neuronal survival was performed. As shown in Fig. 4F, BMSC-CM-induced reduction of apoptosis rate negatively correlated with neuronal survival in HD-intoxicated VSC4.1 cells.

Antibody against NGF was further used to deplete NGF from BMSC-CM. We found that neutralization of NGF abolished the anti-apoptotic efficacy of BMSC-CM in HD-treated VSC4.1 cultures by showing increased number of TUNEL-positive cells and activity of caspase-3 as well as recovered apoptotic appearance (Fig. 4A–G). These results suggest that NGF is indispensible for the anti-apoptotic capacity of BMSC.

### BMSC graft activates Akt pathway and subsequently dissociates the complex of Bad/Bac-xl through Bad phosphorylation

**Figure 5:**
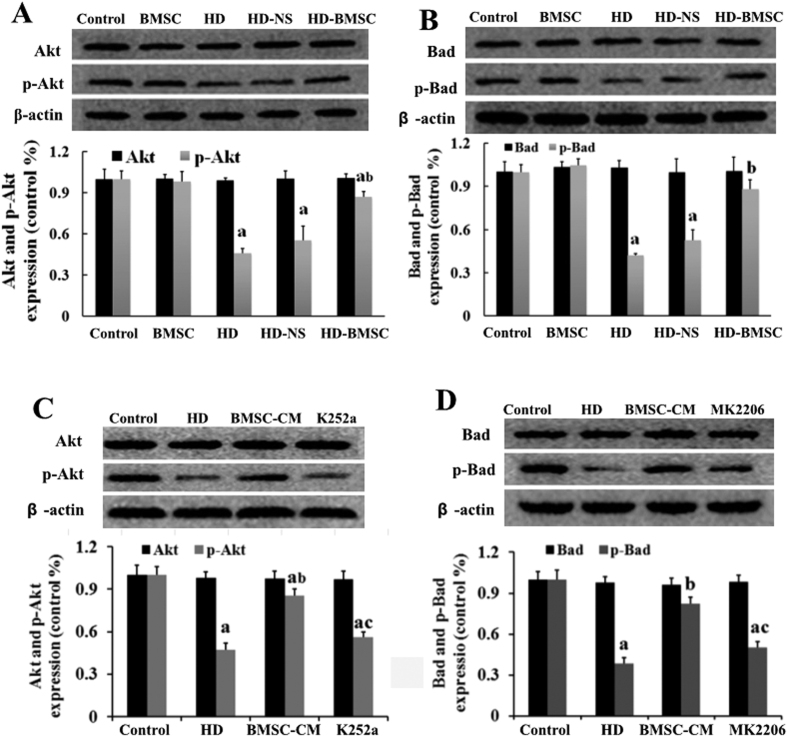
Akt signaling is involved in the anti-apoptotic effect of BMSC. (**A**) The effects of BMSC transplantation on the levels of Akt and p-Akt in the spinal cord of HD-intoxicated rats were detected using Western blot and the density of blots was quantified (the full-length gels were shown in Supplementary Figure 4A–F). (**B**) The effects of BMSCs transplantation on the levels of Bad and p-Bad in the spinal cord of HD-intoxicated rats were detected using Western blot and the density of blots was quantified. (**C,D**) VSC4.1 cells were treated with HD (25 mM) or saline for 24 h and then were treated with BMSC-CM (15%, v/v) in the presence or absence of K252a or MK-2206 1h pre-treatment for additional 24 h. The levels of Akt and p-Akt (**C**) as well as Bad and p-Bad (**D**) were determined using Western blot (the full-length gels were shown in Supplementary Figure 4G–L). ^a^*p* < 0.05, compared with control group; ^b^*p* < 0.05, compared with HD group; ^c^*p* < 0.05, compared with BMSC-CM group.

Akt signaling pathway is an important player in regulating cell survival^22^. To investigate whether Akt pathway is involved in the anti-apoptotic effect of BMSC, the phosphorylation status of Akt was measured. As shown in Fig. 5A, HD intoxication significantly attenuated the level of Akt phosphorylation in the spinal cord of rats, indicating decreased activation of Akt. Interestingly, compared with HD alone group, the suppressed activation of Akt was recovered by BMSC transplantation to the similar level as control rats, indicating that BMSC activate Akt signaling pathway. Bad is a downstream target of Akt and can be phosphorylated by Akt^23^. We subsequently detected the status of Bad phosphorylation. Consistent with Akt activation, the phosphorylation status of Bad was also increased in BMSC-grafted rats compared with HD alone group (Fig. 5B).

To determine whether NGF is involved in BMSC-induced Akt activation, k252a, an inhibitor of TrkA receptor was used. As seen in Fig. 5C, BMSC-CM-induced elevation of Akt phosphorylation in HD-intoxicated VSC4.1 cultures was significantly decreased in the presence of K252a. To clarify BMSC-induced Bad phosphorylation was dependent on Akt, the status of Bad phosphorylation in BMSC-CM-treated VSC4.1 cells with HD intoxication was further examined in the presence of Akt inhibitior, MK-2206. As shown in Fig. 5D, MK-2206 significantly mitigated the elevation of Bad phosphorylation induced by BMSC-CM, indicating that BMSC-induced Bad phosphorylation is Akt-dependent.

**Figure 6:**
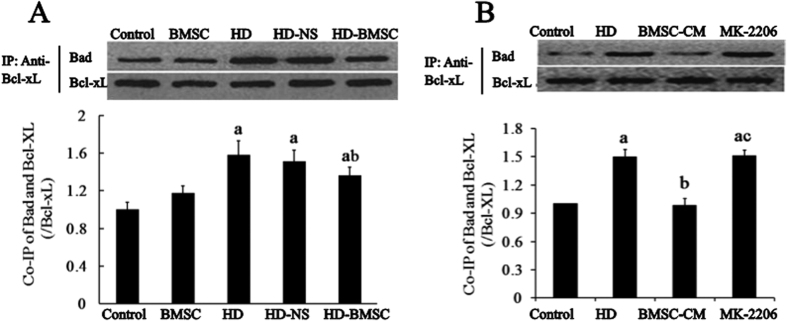
The dissociation of Bad/Bcl-xl complex is Akt-dependent after BMSC treatment. (**A**) Co-IP was used to detect the effect of BMSC transplantation on the dissociation of Bad/Bcl-xl complex in spinal cordof HD-intoxicated rats. (**B**) The interaction of Bad and Bcl-xl in BMSC/HD-exposed VSC4.1 cells with or without MK-2206 was detected by Co-IP. ^a^*p* < 0.05, compared with control group; ^b^*p* < 0.05, compared with HD group; ^c^*p* < 0.05, compared with BMSC-CM group.

Bad phosphorylation is known to enhance the dissociation of Bad/Bcl-xL complex^24^. To seek the direct evidence of dissociation of Bad/Bcl-xL complex after BMSC treatment, co-immunoprecipitation experiments were performed. We found an increased level of Bad co-precipitated by anti-Bcl-xL antibody in the spinal cords of HD-intoxicated rats, which was significantly decreased by BMSC transplantation, indicating the dissociation of Bad/Bcl-xL complex by BMSC (Fig. 6A). A similar finding was observed in BMSC-CM/HD-treated VSC4.1 cells compared with HD alone group (Fig. 6B).

To determine whether the increased dissociation Bad/Bcl-xL complex by BMSC was dependent on Akt, co-immunoprecipitation experiment was performed in the presence of Akt inhibitor, MK-2206. We found that the decreased level of Bad co-precipitated by anti-Bcl-xL antibody in BMSC-CM/HD-treated VSC4.1 cells was recovered by MK-2206 treatment (Fig. 6B), indicating Akt-dependent manner.

### Inhibition of Akt abolishes the anti-apoptotic effect of NGF

**Figure 7:**
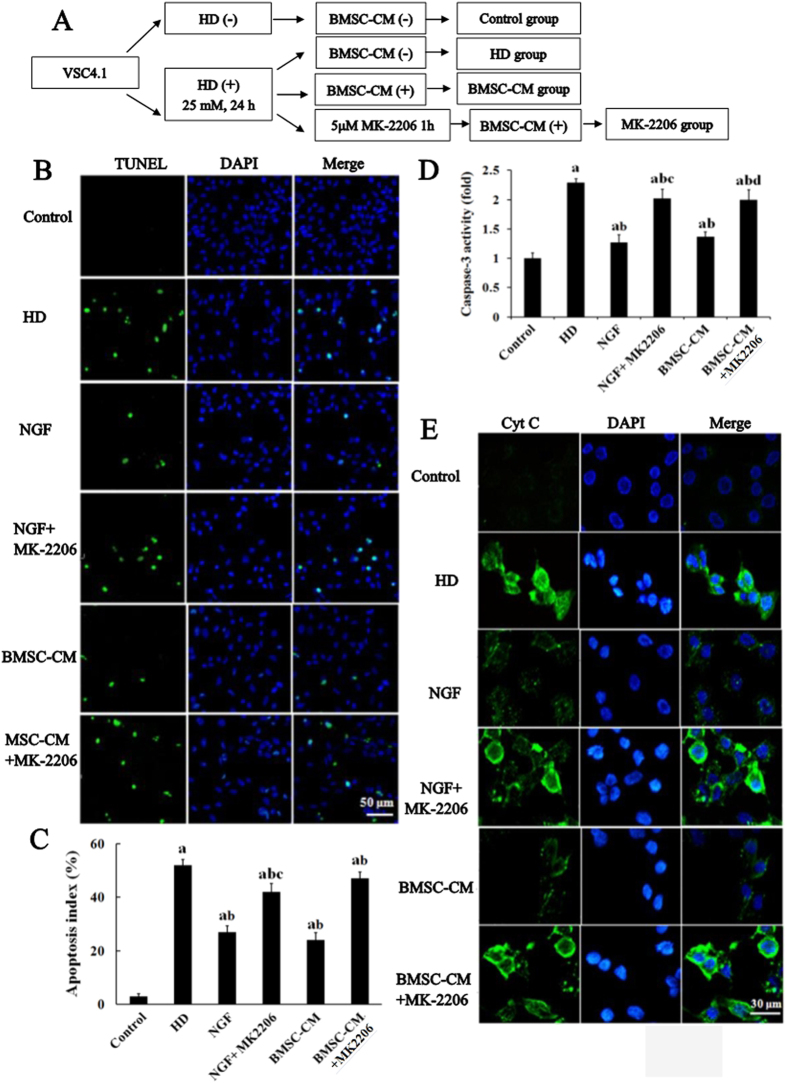
Inhibition of Akt abolishes the anti-apoptotic effects of BMSC and NGF. (**A**) Schematic diagram of experimental design. VSC4.1 cells were treated with HD (25 mM) or saline for 24 h and then were treated with BMSC-CM (15%, v/v) or NGF (100 μM) in the presence or absence of MK-2206 1 h pre-treatment for additional 24 h. (**B**) TUNEL and DAPI staining were performed to detect apoptosis of cellsin different groups. (**C**) The percentage of TUNEL-positive cells in different groups was quantified. (**D**) The activity of caspase-3 in different groups was detected using commercialcaspase-3 activity detection kit. (**E**) Cyt C was stained in different groups and the representative images were shown. ^a^*p* < 0.05, compared with control group; ^b^*p* < 0.05, compared with HD group; ^c^*p* < 0.05, compared with BMSC-CM group.

The central role of Akt signaling in the anti-apoptotic effect of BMSC prompted us to test whether inhibition of Akt was capable of blocking BMSC-enhanced neuron survival. HD-intoxicated VSC4.1 cells were treated with Akt inhibitor MK-2206, prior to BMSC-CM or NGF treatment (Fig. 7A). As shown in Fig. 7B,C, BMSC-CM or NGF treatment reduced the number of TUNEL-positive cells in HD-intoxicated VSC4.1 culture. In contrast, in the presence of MK-2206, BMSC-CM and NGF failed to interfere with HD-induced apoptosis in VSC4.1 cells, indicating that Akt inhibition blocked the anti-apoptotic capacity of BMSC.

In addition to TUNEL staining, the activity of caspase-3 and Cyt C expression were also examined with or without MK-2206 treatment. Consistent with that of TUNEL staining, decreased caspase-3 activity and Cyt C expression in HD-treated VSC4.1 cultures by BMSC-CM or NGF treatment were markedly attenuated in the presence of MK-2206 (Fig. 7C,D). These results suggest that inhibition of Akt abolishes the neuroprotective effect of BMSC against HD-induced apoptosis.

## Discussion

In this study, we demonstrated that BMSC transplantation exerts potent anti-apoptotic and neuroprotective effects in a rat model of HD-induced neuropathy. The salient features of our findings are: (1) BMSC transplantation attenuated HD-induced neuron apoptosis in the spinal cord of rats; (2) NGF was a key factor responsible for the anti-apoptotic capacity of BMSC; (3) BMSC activated Akt/Bad signaling pathway in a NGF/TrkA-dependent manner, resulting in dissociation of Bad/Bcl-xL complex; (4) Pharmacological inhibition of Akt abolished the anti-apoptotic capacity of BMSC and NGF in HD-generated neurotoxic model *in vitro*.

Apoptosis is a necessary physiological process that maintains the stability and environmental adaptation of neurons in both developmental and adult nervous system. The disruption of this process is associated with a wide variety of neurodegenerative diseases, including HD-induced neuropathy^25^. Previously, we reported the protective effect of BMSC-CM against HD-induced apoptosis in PC12 cells, which was associated with reduction of Bax expression, Cyt C release and caspase-3 activity^9^. In this study, we delve deeper into investigating the anti-apoptotic and neuroprotective efficacy of BMSC in HD-intoxicated rats. We found that BMSC transplantation performed after 5 weeks of HD intoxication potently ameliorated neuronal apoptosis and reversed the release of Cyt C from mitochondria to cytosol and caspase-3 activity in spinal cord of rats. The high efficacy with a post-treatment regimen suggests that BMSC transplantation may be a promising solution to slow or even halt HD-induced neuropathy.

Mechanistically, the most critical question to address is why BMSC transplantation after onset of neuronal damage still displays protection. BMSC are known to be capable of secreting neurotrophic factors including NGF, which plays a central role in the beneficial effects of BMSC^26^,^27^. Our findings are consistent with previous reports by showing that BMSC transplantation increased the expression of NGF and activation of NGF receptor, TrkA. These results prompted us to further explore the downstream signaling pathways responsible for the anti-apoptotic effect of BMSC. Akt is a type of cellular protein kinase, whose activation is involved in combating apoptosis and supporting neuron survival^28^. Fu *et al.* reported that activation of Akt by piceatannol suppressed Bcl-2 expression and caspase-3/9 activities, thereby protected PC12 cells against β-amyloid-induced apoptosis^29^. Consistently, inhibition of Akt signaling pathway resulted in apoptosis in cultured cerebellar granule cells^30^. Combating apoptosis by Akt activation was also reported in Wang’s study, in which carbenoxolone-afforded neuroprotection against ischemia injury-induced apoptosis was blocked by PI3K/Akt inhibitor, LY294002^31^. However, whether Akt signaling pathway is involved in BMSC against HD-induced neuronal apoptosis remains unknown. Our results showed that BMSC transplantation induced activation of Akt, being consistent with previous reports. Neutralization of NGF or inhibition of TrkA markedly decreased the activation of Akt induced by BMSC-CM in HD-treated VSC4.1 cells, suggesting that Akt is the downstream pathway of NGF. The importance of Akt pathway was further corroborated by showing that pharmacological inhibition of Akt markedly abrogated the anti-apoptotic capacity of BMSC-CM against HD-induced neurotoxicity.

Akt activation can phosphorylate downstream target Bad. Phosphorylated Bad binds cytosolic protein 14-3-3 to release anti-apoptotic protein Bcl-xL, which can block the mitochondrial translocation of pro-apoptotic protein Bax, maintain mitochondrial membrane potential (MMP) and prevent the release of Cyt C from the mitochondria as well as cell apoptosis^32^,^33^,^34^. Consistent with Akt activation in HD-intoxicated rats with BMSC transplantation, BMSC also increased the level of Bad phosphorylation in the spinal cord (Fig. 5). Inhibitor of Akt attenuated BMSC-CM-elevated Bad phosphorylation, indicating that BMSC-induced Bad phosphorylation is mediated by Akt pathway. Further co-immunoprecipitation study in spinal cord of HD-intoxicated rats with BMSC transplantation and in BMSC-CM/HD-treated VSC4.1 cells revealed the increased dissociation of the proapoptotic complex of Bad/Bcl-xL in the mitochondrial fraction by showing a decreased level of Bad that binds to Bcl-xL. Our data suggest that Akt activation and subsequent Bad phosphorylation is involved in the anti-apoptotic function of BMSC.

Due to BMSC are easily to obtain and expand *in vitro* as well as they display neurotrophic and anti-apoptotic properties, BMSC therapy gains popularity among practitioners and researchers. Currently, multiple modes of administration exist for stem cell transplantation in experimental animals and human clinical trials, including intrathecal, intravenous and intraspinal^35^,^36^,^37^. Several studies reported that intravenous injection of BMSC is a relative easy way in clinic for patients to receive transplantation without any risk for further damage^37^,^38^. In addition, BMSC intravenous injection is a relatively safe procedure and has no side effects, such as tumor development and sustained immune dysregulation, up to 26 weeks after transplantation in experimental animals and as long as 12 months in patients^37^,^39^. Therefore, in the present study, the demonstration of high efficacy in neuroprotection by intravenous BMSC transplantation with a post-treatment regimen is highly significant from clinic point of view.

In summary, this study provides convincing evidence that BMSC transplantation potently attenuated neuronal apoptosis, Cyt C release and caspase-3 activity induced by HD in the spinal cord of rats through a NGF/Akt-dependent manner, even performed after 5 weeks of HD intoxication. The potent protective effects even treated after onset of neuronal damage suggest that BMSC transplantation could be a promising candidate for combating *n*-hexane-induced neuropathy. Future studies focusing on the recovery of pathology and motor functions by BMSC in animal model of *n*-hexane-induced neuropathy should be guaranteed.

## Material and Methods

### BMSC culture

BMSC were isolated from the femurs and tibias of SD rats (Laboratory Animal center, Dalian Medical University, China) as previously described^40^. Briefly, rats (60~90 g) were euthanatized by cervical dislocation and bone marrow was flushed with PBS. A single-cell suspension was obtained by sieving through 70-μm nylon gauze. Cells were then seeded in Dulbecco’s modified eagle medium-low glucose (DMEM-LG, Gibco, USA) with 10% fetal bovine serum (FBS, HyClone, USA), 100 U/ml penicillin and 100 μg/ml streptomycin (Beyotime, China) at 37 °C in 95% humidified air. Non-adherent cells were removed after 24 h by replacing the media. The morphological features and characteristic surface makers (CD29, CD45 and CD90) detected by flow cytometry were used to identify MSCs. Adipogenic and osteogenic differentiation ability of MSCs was also evaluated after induction by specific media Oil Red O and Alizarin Red S staining (Cyagen, China). All the experiments were done within 5th passage.

### Preparation of BMSC-CM

Confluent BMSC, at 3~5 passage, were washed with phosphate-buffered saline (PBS) and transferred to DMEM with 10% FBS for 24 h. The culture supernatants were collected and then centrifuged at 1,500 rpm for 10 minutes at 4 °C. The supernatants were transferred to a centrifugal column with a 3 kDa cut-off (Millipore, Billerica, MA, USA) and centrifuged at 3,500 rpm for 45 minutes at 4 °C. The concentrated BMSC-CM was desalted according to the manufacturer’s protocol and then was sterilized by filtration with 0.22 μm membrane.

### Animal treatment and tissue preparation

Fifty adult male SD rats (200∼230 g) were purchased from the Experimental Animal Center of Dalian Medical University. The rats were housed in polycarbonate boxes, with sufficient drinking water and food. The animal room was maintained at approximately 22 °C and 50% relative humidity with a 12 h light-dark cycle. They were housed in the Experimental Animal Center of Dalian Medical University for 7 days for acclimatization. Fifty rats were randomly divided into 5 groups (n = 10 for each group). Rats were intoxicated with HD by intraperitoneal injection (i.p.) at dosages of 400 mg/kg/day (five times per week), according to Torres *et al.*^41^ and Cui *et al*’s reports^6^. HD was dissolved in 0.9% saline and administered at 3 ml/kg body weight/dose. The corresponding control group rats received an equivalent volume of 0.9% saline by i.p.

On completion of 5 weeks of HD administration, rats were transplanted with BMSC (5 × 10^7^cells/kg) by tail vein injection. Additional 5 weeks later, rats were killed by cervical decapitation. The spinal cords were quickly dissected and frozen in liquid nitrogen before storing them at −80 °C. Experiments were performed in accordance with the Animal Guideline of Dalian Medical University. All experimental protocols were approved by and in agreement with the Ethical Committee of Dalian Medical University.

### Neurological testing

Rats were placed in an open place and observed for 3 min^14^,^17^. A trained, test-blinded observer who was not involved in animal care or HD exposure performed the behavioral evaluation. A gait score was assigned from 1 to 4, where 1: a normal, unaffected gait; 2: a slightly affected gait (tip-toe walking, slight ataxia, and hindlimb weakness); 3: a moderately affected gait (obvious movement abnormalities characterized by dropped hocks and tail dragging); and 4: a severely affected gait (frank hindlimb weakness and inability to rear). Three successive measurements were averaged for each HD-intoxicated or control rats^42^.

### Electron microscope (SEM) analysis

Fixed spinal cord were dehydrated gradiently, embedded, and then sliced with an ultramicrotome, as previously described^13^,^15^,^16^. These specimens were stained with uranyl acetate and lead citrate. Transmission electron microscope (H/7500, Hitachi, Japan) was utilized to observe the pathological changes of the axon.

### VSC4.1 cell culture

VSC4.1 motor neurons were maintained in DMEM medium containing 15 mM HEPES, pyridoxine NaHCO_3_ (Sigma, St. Louis, Missouri, USA), 2% Sato’s components, 1% penicillin and streptomycin (Beyotime, Shanghai, China) as well as 15% heat-inactivated fetal bovine serum (Hyclone, Logan, UT, USA) in poly-L-ornithine coated 75 cm^2^ flasks. Cells were grown at 37 °C in the incubator with 5% CO_2_ and full humidity.

### TUNEL assay

TUNEL assays were performed in the spinal cords of rats (10 μm sections) and VSC4.1 cells by using Roche’s *In Situ* Cell Death Detection Kit (Roche, Mannheim, Germany) according to the manufacturer instructions. DAPI was used to counter stain the nuclei. Cells were observed using a fluorescence microscope (×400 magnification). Six fields were randomly selected and the percentage of positive cells was calculated as the apoptosis index (AI) using the following equation: AI = (number of positive cells/total number of cells) × 100%. The quantification of TUNEL staining was performed by using 10 representative photos each rat and results are expressed as a percentage of vehicle controls (mean ± SEM).

### Determination of caspase-3 activity

The activity of caspase-3 was determined using the Caspase-3 activity detection kit (Beyotime, Shanghai, China) according to the manufacturer’s protocol. Briefly, the spinal cords were homogenized using the lysis buffer supplied by the kit. Assay was performed on 96-well microtitre plates by incubating 40 μg protein of lysate per sample in 50 μl reaction buffer (1% NP-40, 20 mM Tris-HCl (pH 7.5), 137 mM Nad and 10% glycerol) containing 10 μl caspase-3 substrate (Ac-DEVD-pNA) (2 mM) at 37 °C for 2 hours. The absorption at 405 nm was measured. The data in other experiments were obtained from three independent experiments with triplication.

### Western blot

Tissue and cells were homogenized in ice-cold RIPA Tissue Protein Extraction Reagent (Beyotime, China) supplemented with 1% proteinase inhibitor mix. The proteins were separated by SDS-PAGE and then electrotransferred to PVDF membrane (Millipore, France). The membrane was incubated with appropriate primary antibodies overnight at 4 °C. Antibodies used were NGF (1:500, Cell Signaling Technology, USA), TrkA (1:1000, sigma, USA), p-TrkA (1:1000, sigma, USA), Akt (1:1000, sigma, USA), p-Akt (ser-473) (1:1000, sigma, USA), Bad (1:1000, sigma, USA), p-Bad (ser-136)(1:1000, sigma, USA), Cyt C (1:500, Beyotime, China), VDAC (1:500, Abcam, USA), β-actin (1:500, ZS-Bio, China). Immunoreactivity was visualized by second horseradish peroxidase-conjugated antibody (1:5000, Sigma, USA) and enhanced chemoluminescence (Beyotime, China). Quantified densitometric analysis was performed with UVP BioSpectrum Multispectral Imaging System (Ultra-Violet Products Ltd. USA). The quantification of WB were obtained from three independent experiments with triplication.

### Confocal double-label immunofluorescence

The frozen sections (10 μm) of spinal cords were immunoblocked with 10% donkey serum in 5% BSA for 1h and then incubated with mouse monoclone anti-Cyt C antibody (1:200), overnight at 4 °C. On the second day, the sections were washed by PBS for 3 times before incubation with polyclonal rabbit anti-MAP-2 antibody (1:500) overnight at 4 °C. The double-label immunofluorescence pictures were taken under the confocal microscope by using Alexa-488 (green) and Alexa-594 (red) conjugated secondary antibodies (1:500).

### Co-staining Tunel and immunofluorescence

The frozen sections (10 μm) of spinal cords were immunoblocked with 10% donkey serum in 5% BSA for 1h and then incubated with monoclone anti-MAP-2 antibody (1:200), anti-GFAP antibody (1:500), anti-Iba-1 antibody (1:500), respectively, overnight at 4 °C. On the second day, the sections were conjugated secondary antibodies, then performed TUNEL assays according to the manufacturer instructions. The pictures were taken under the confocal microscope by using Alexa-488 (green) and Alexa-594 (red).

### Quantitative real-time PCR analysis

Total RNA was extracted from the spinal cords using RNAiso Plus (Takara, Tokyo, Japan) according to the manufacturer’s instructions. One microgram of RNA was reverse transcribed using a Reverse Transcription Kit (Takara, Tokyo, Japan) in T100™ Thermal Cycler (Bio-rad, Hercules, USA). Real-time Q-PCR was performed with a SYBR Green PCR kit (Takara, Tokyo, Japan) using the TP800 Real-Time PCR Detection System (Takara, Tokyo, Japan). The following primer pairs were used (Takara, Dalian): NGF, 5′-TGC CCC TGC TGA ACC AA-3′/5′-GCT TGC TCC TGT GAG TCC TGT-3′; β-actin, 5′-GGA GAT TAC TGC CCT GGC TCC TA-3′/5′-GAC TCA TCG TAC TCC TGC TTG CTG-3′ (designed by Takara, Dalian, China). The reaction conditions were 95 °C for 30 s, 55 °C for 30 s, and 72 °C for 30 s for 40 cycles. All the data were normalized to β-actin.

### Coimmunoprecipitation

The spinal cords of rats were collected and mitochondrial fraction was extracted. Approximately 100 μg mitochondrial fraction was used for coimmunoprecipitation. The protein sample was mixed with 20 μl protein A+G sepharose (Beyotime, Shanghai, China) and incubated for 30 min at 4 °C, and then the samples were centrifuged at 1,200 × g for 10 min. The supernatant was incubated with 2 μg polyclonal rabbit anti-Bcl-xL antibody (1:1000) and 15 μl of protein A+G sepharose (50% slurry) for 5 h at 4 °C. Protein A + G beads were collected by centrifugation at 1,200 × g for 5 min and were washed 6 times with TNE buffer (10 mM Tris-HCl at pH 7.5, 1% NP-40, 0.15 M NaCl, 1 mM EDTA and 1:100 protease inhibitor cocktail). Bound proteins were eluted in sample buffer, separated on 4–12% SDS-PAGE, and blotted with anti-Bad and anti-Bcl-xL antibodies.

### Statistical analysis

All results were expressed as mean ± S.D., and the statistical analysis was performed with one-way analysis of variance (ANOVA), followed by LSD test, which was performed using SPSS 13.0 statistical software. The p-values less than 0.05 were considered to be significant.

## Additional Information

**How to cite this article**: Wang, Q. *et al.* Bone marrow mesenchymal stem cells attenuate 2,5-hexanedione-induced neuronal apoptosis through a NGF/AKT-dependent pathway. *Sci. Rep.* **6**, 34715; doi: 10.1038/srep34715 (2016).

## Supplementary Material

Supplementary Information
