# Supplementary material for: Bone marrow mesenchymal stem cells attenuate 2,5-hexanedione-induced neuronal apoptosis through a NGF/AKT-dependent pathway
Source: Sci Rep. 2016 Oct 5;6:34715. doi: 10.1038/srep34715 (PMC5050456; doi:10.1038/srep34715)
Supplement: Supplementary Information [file srep34715-s1.pdf]

Bone marrow mesenchymal stem cells attenuate 2,5-hexanedione-induced neuronal apoptosis through a NGF/AKT-dependent pathway

Qingshan Wang[1*], Guohua Sun[2*], Chenxue Gao[1], Lina Feng[1], Yan Zhang[1], Jie Hao[1], Enjun Zuo[3], Cong Zhang[4], Shuangyue Li[1#], Fengyuan Piao[1#]

[1] Department of Occupational and Environmental Health, Dalian Medical University, Dalian, Liaoning 116044, China

[2] Department of Clinical Laboratory, the First Affiliated Hospital of Dalian Medical University, Liaoning 116011, China

[3] College of Stomatology, Dalian Medical University, Dalian, Liaoning 116044, China

[4] Department of Nutrition and Food Safety, Dalian Medical University, Dalian, Liaoning 116044, China

* Qingshan Wang and Guohua Sun as co-first author contributed equally to this work.

#Corresponding authors: Fengyuan Piao E-mail: piaofengyuan353@163.com ; Shuangyue Li E-mail: lsy236@163.com

Abstract

Growing evidence suggests that the increased neuronal apoptosis is involved in *n*-hexane-induced neuropathy. We have recently reported that bone marrow-mesenchymal stem cells-derived conditioned medium (BMSC-CM) attenuated 2,5-hexanedione (HD, the active metabolite of *n*-hexane)-induced apoptosis in PC12 cells. Here, we explored the anti-apoptotic efficacy of BMSC *in vivo*. HD-treated rats received BMSC by tail vein injection 5 weeks after HD intoxication. We found that in grafted rats, BMSC significantly attenuated HD-induced neuronal apoptosis in the spinal cord, which was associated with elevation of nerve growth factor (NGF). Neutralization of NGF in BMSC-CM blocked the protection against HD-induced apoptosis in VSC4.1 cells, suggesting that NGF is essential for BMSC-afforded anti-apoptosis. Mechanistically, we found that the decreased activation of Akt induced by HD was significantly recovered in the spinal cord by BMSC and in VSC4.1 cells by BMSC-CM in a TrkA-dependent manner, leading to dissociation of Bad/Bcl-xL complex in mitochondria and release of anti-apoptotic Bcl-xL. The importance of Akt was further corroborated by showing the reduced anti-apoptotic potency of BMSC in HD-intoxicated VSC4.1 cells in the presence of Akt inhibitor, MK-2206. Thus, our findings show that BMSC attenuated HD-induced neuronal apoptosis *in vivo* through a NGF/Akt-dependent manner, providing a novel solution against *n*-hexane-induced neurotoxicity.

Key words 2,5-hexanedione, neurotoxicity, Apoptosis, bone marrow-mesenchymal stem cells

A 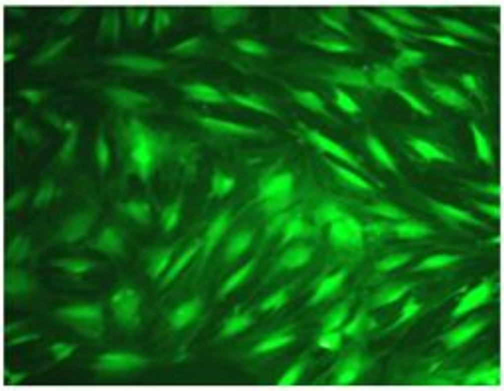 B 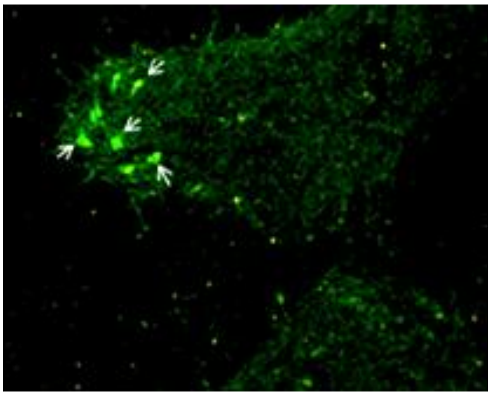

Supplementary Figure 1. (A) BMSC were labeled by CFSE before graft. Incubation with 10 μmol/L CFSE for 15 mins, over 98% BMSC was labeled. (B) CFSE-labeled BMSCs were detected in the spinal cord of HD-intoxicated rat after BMSC transplantation 5 weeks.

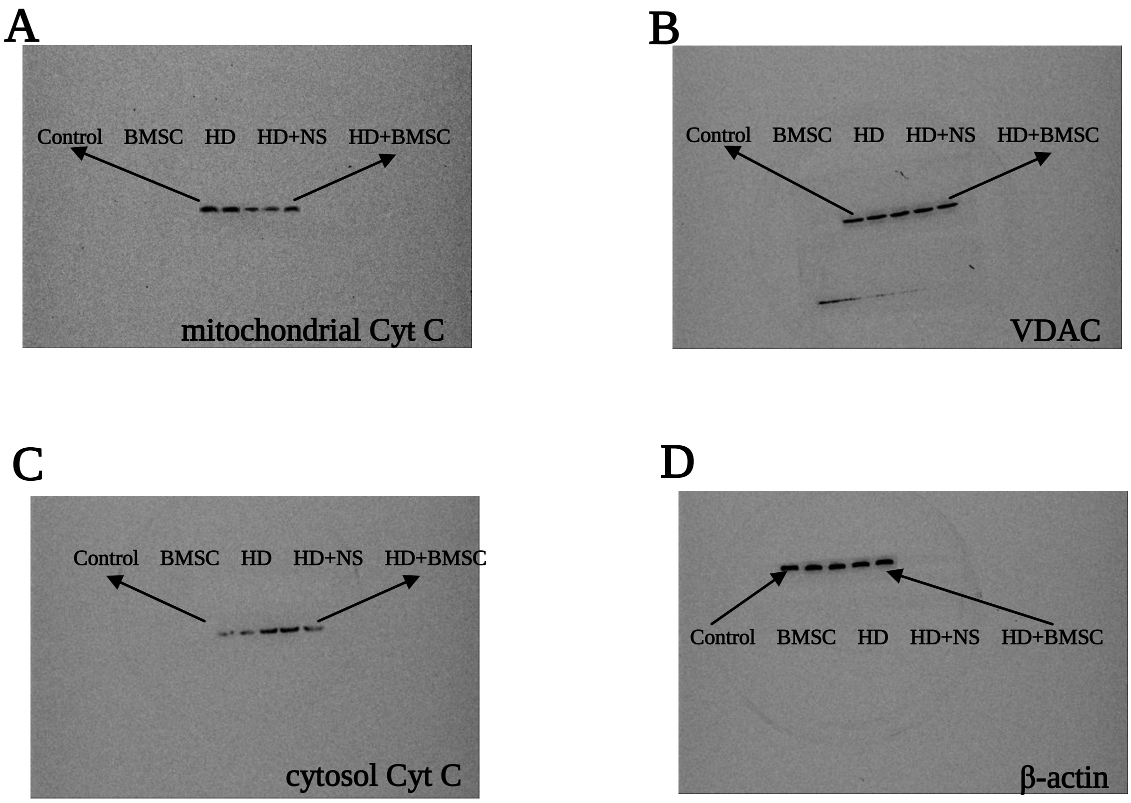

Supplementary Figure 2. The full-length gels for Figure 1C. The full-length Western blot gels of mitochondrial Cyt C and its VDAC (A, B) and cytosol Cyt C and its β-actin (C, D) in the spinal cord of HD-intoxicated rats with or without BMSC transplantation.

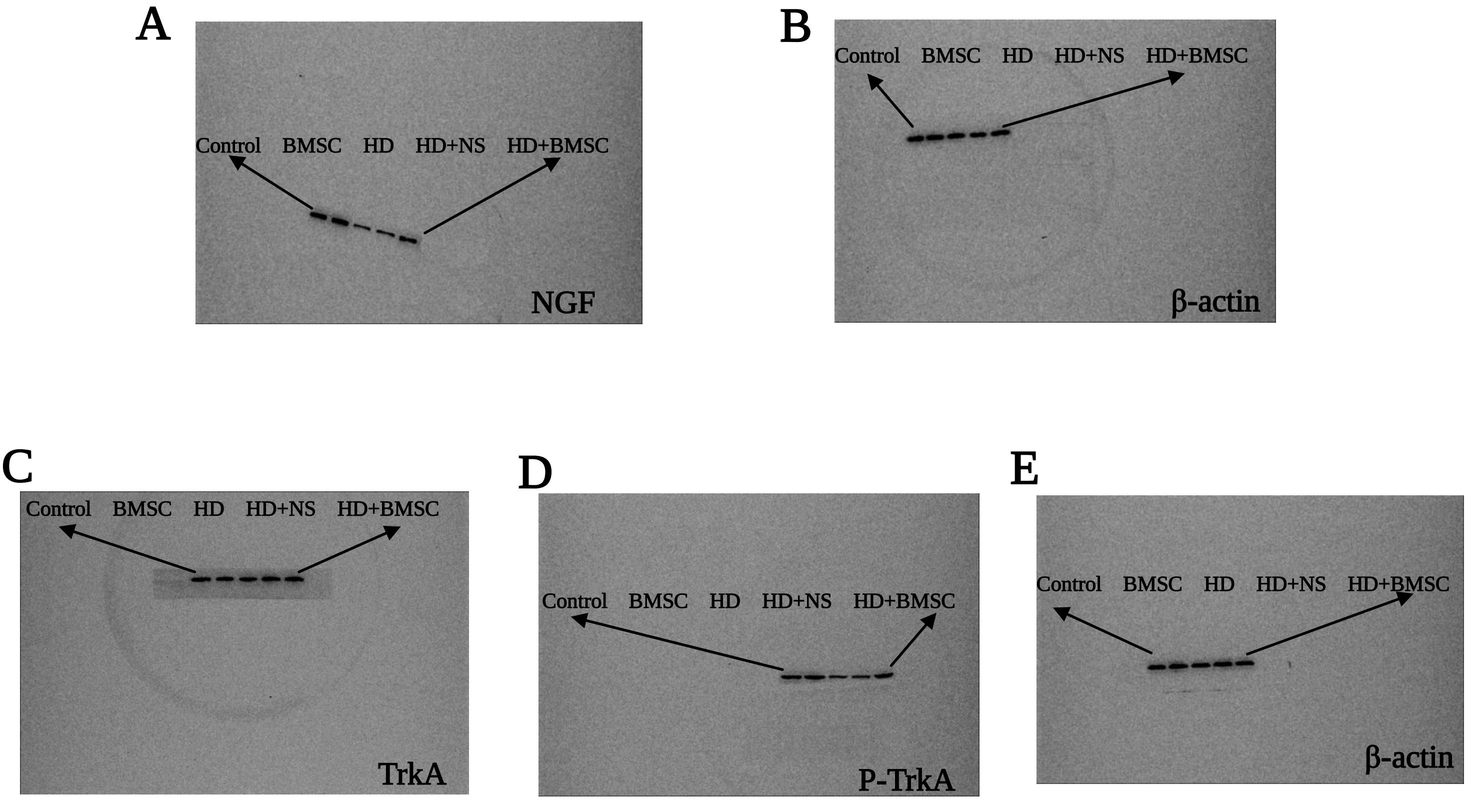

Supplementary Figure 3. The full-length gels for Figure 3. (A, B) The full-length Western blot gels of NGF and β-actin in the spinal cord of HD-intoxicated rats with or without BMSC transplantation; (C-E) The full-length Western blot gels of TrkA, p-TrkA and β-actin in the spinal cord of HD-intoxicated rats with or without BMSC transplantation.

`

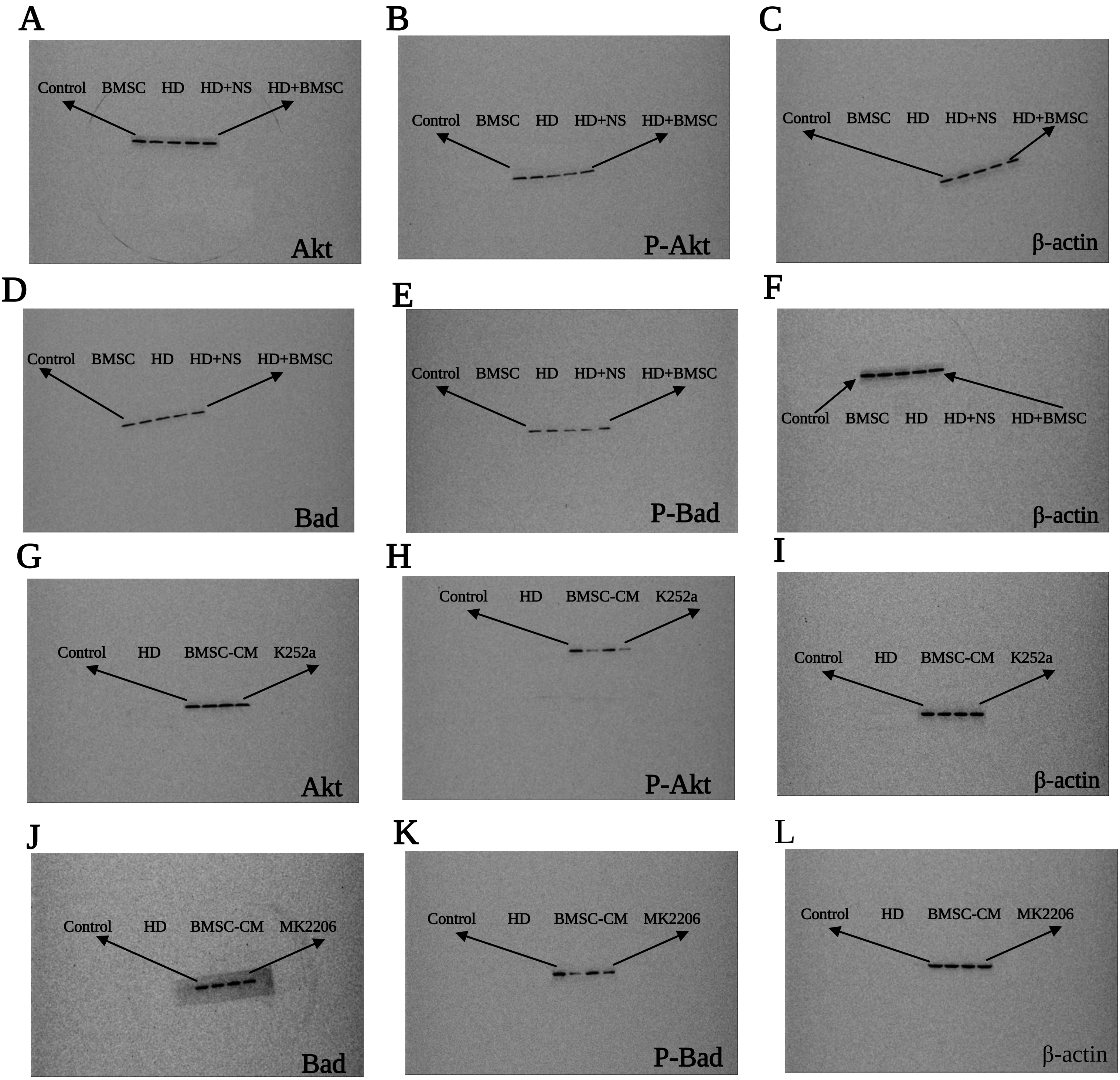

Supplementary Figure 4. The full-length gels for Figure 5. (A-C) The full-length Western blot gels of Akt, p-Akt and β-actin in the spinal cord of HD-intoxicated rats with or without BMSC transplantation; (D-F) The full-length Western blot gels of Bad, p-Bad and β-actin in the spinal cord of HD-intoxicated rats with or without BMSC

transplantation; (G-I) The full-length Western blot gels of Akt, p-Akt and β-actin in HD-intoxicated VSC4.1 cells with or without K252a treatment; (J-L) The full-length Western blot gels of Bad, p-Bad and β-actin in HD-intoxicated VSC4.1 cells with or without MK2206 treatment.
